# A Judge on Medical Witnesses' Fees

**Published:** 1914-09-12

**Authors:** 


### A Judge on Medical Witnesses' Fees.

In an account of costs in a workman's compensation action the fees which the auditor allowed to the medical men who attended as skilled witnesses were objected to as being excessive, the argument in support of the objection being that the doctors had appeared in two cases on the same day. In repelling the objection the judge remarked: "It would be unfortunate if a parsimonious policy in respect to the fees allowed to professional witnesses resulted in preventing the attendance of men of authority and standing, and it would not lead to economy if these witnesses were not encouraged to make it possible by waiting sometimes for many hours to hold two or more arbitrations on the same day. In this Court the days available for arbitration are very limited, and medical men are often put to great inconvenience in waiting the turn of the particular case in which they have to give evidence. It is only by the occasional fortune of being engaged in more than one on the same day that their remuneration approaches generosity."

